# Identification of group B respiratory syncytial viruses that lack the 60‐nucleotide duplication after six consecutive epidemics of total BA dominance at coastal Kenya

**DOI:** 10.1111/irv.12131

**Published:** 2013-06-20

**Authors:** Charles N. Agoti, Caroline W. Gitahi, Graham F. Medley, Patricia A. Cane, D. James Nokes

**Affiliations:** ^1^ Centre for Geographic Medicine Research – CoastKenya Medical Research Institute (KEMRI)KilifiKenya; ^2^ School of life SciencesUniversity of WarwickCoventryUK; ^3^ Public Health EnglandLondonUK

**Keywords:** Attachment (G gene), BA genotype, genetic diversity, respiratory syncytial virus

## Abstract

Respiratory syncytial virus BA genotype has reportedly replaced other group B genotypes worldwide. We report the observation of three group B viruses, all identical in G sequence but lacking the BA duplication, at a coastal district hospital in Kenya in early 2012. This follows a period of six consecutive respiratory syncytial virus (RSV) epidemics with 100% BA dominance among group B isolates. The new strains appear only distantly related to BA variants and to previously circulating SAB1 viruses last seen in the district in 2005, suggesting that they were circulating elsewhere undetected. These results are of relevance to an understanding of RSV persistence.

## Background

Human respiratory syncytial virus (RSV) is the leading viral cause of acute lower respiratory tract illnesses in infants and young children worldwide^1^ and is associated with annual or biannual epidemics.^2^ RSV isolates can be classified into two genetically and antigenically distinct groups, A and B, within which further diversity is reported.^3^ RSV repeatedly re‐infects individuals throughout life, and the strains involved are often genetically distinct.^4^ The differences occurring both between and within the groups are most pronounced at the attachment (G) protein gene, which encodes a surface‐expressed protein of the virus known to be targeted by the host neutralizing antibody response.^1^

In the last two decades, sequencing and phylogenetic analysis of the G gene have allowed identification of several genotypes within the two groups.^3^ It has also been noted that the circulating diversity within the groups is dynamic, and new genotypes periodically emerge, but also some previously circulating genotypes appear to have become extinct.^3^ Locally, over successive epidemics, predominant genetic variants within the groups become replaced.^3^ Continued observation of the dynamics of circulating RSV genetic and antigenic diversity is essential for the design of better RSV control approaches.

A new group B strain with a 60‐nucleotide duplication within the G gene was first observed in Buenos Aires, Argentina, in 1999 (named BA genotype) and spread around the world in 3–4 years.^5^ Interestingly, the BA genotype appeared to replace all the other group B genotypes in RSV epidemics that occurred in the second half of the last decade.^6^ The cause of the remarkable BA genotype epidemiological success remains unknown.^7^ Few non‐BA cases were observed after 2005,^8^, ^9^, ^10^ these occurring in Cambodia, Brazil and China between 2007 and 2009. The epidemiological dynamics of the BA genotype have indicated that (i) new RSV strains can transmit to all parts of the world in a relatively short time period, and (ii) there is ongoing within‐group competition between RSV genotypes. Monitoring the transmission and evolutionary dynamics of group B RSV strains after the BA genotype emergence allow the study of an RSV genotype from emergence to, potentially, its extinction.

Hospital‐based RSV surveillance at coastal Kenya, 2002–to date, was established with the objectives of documenting RSV disease burden and RSV molecular and immuno‐epidemiology^11^ within the region. The surveillance period overlaps with the period when changes have been observed in circulating RSV B genotypes around the world. Here, we report the results from sequencing and genotyping of all group B RSVs that we identified over the 11 years of the surveillance, and the results of analysis of novel non‐BA strains we observed in three patients infected in early 2012.

## Methods

The samples analyzed were obtained from under five‐year‐olds admitted with pneumonia to the Kilifi District Hospital^11^ and span the period between January 2002 and mid‐2012 (the latter marking the end of the 2011–2012 epidemic). Either a parent or a guardian provided informed consent on behalf of each child. The specimens (nasal washings, aspirates, or swabs) were screened for RSV using an immunofluorescence assay (IFAT), and samples positive for RSV were subtyped into groups A and B using a conventional multiplex RT‐PCR or a multiplex real‐time PCR assay. Group B RSV‐positive samples were sequenced in the ectodomain region of the G gene as previously described.^4^

Sequences were aligned using bioedit software (http://www.mbio.ncsu.edu/bioedit/bioedit.html). Phylogenetic analyses were performed in mega 5.1 program (http://www.megasoftware.net/). The dates to the most recent common ancestor (MRCA) between sequence clusters were estimated using Bayesian methods within the beast software version 1.74,^12^ and convergence was confirmed in tracer program version 1.5. Representative strains of previously identified group B genotypes were included in the analysis to help classify the Kilifi strains.

## Results and discussion

Over the period January 1, 2002, to June 31, 2012, 574 group B RSVs were identified among the RSV IFAT‐positive samples, and 488 (85·0%) of these were successfully sequenced in the G ectodomain region. The genotyping results over the 11 consecutive epidemics are summarized in Table 1. Early in the surveillance, BA strains were detected as a rare genotype, making only 7·3% (3 of 41) of the sequenced group B genotypes between January 2002 and June 2004. The prevalence of BA strains dramatically increased to 97·3% (108 of 111) in the 2004–2005 epidemic, and thereafter, all the group B samples identified and sequenced between June 2005 and December 2011 possessed the BA duplication. However, in sequencing B specimens from the first half of 2012, three lacked the BA duplication. This was an unexpected finding occurring after multiple epidemics of non‐BA genotypes absence; thus, we undertook further analysis of these three sequences to ascertain their possible origin and phylogenetic relationship with other RSV sequences deposited in public databases.

**Table 1:** The distribution of the different group B genotypes at Kilifi during the 11 consecutive epidemics of the surveillance

Peak epidemic month	Total group B strains identified	No. group B sequenced (%)^a^	Genotypes identified among the sequenced group B strains		
			SAB1	BA (%)^b^	New type
May 2002	1	1 (100·0)	1	0 (0)	0
Jan 2003	33	27 (81·8)	25	2 (7·4)	0
Apr 2004	18	13 (72·2)	12	1 (8·3)	0
Jan 2005	117	111 (94·9)	3	108 (97·3)	0
Mar 2006	9	9 (100·0)	0	9 (100)	0
Jan 2007	22	19 (86·2)	0	19 (100)	0
Jan 2008	158	140 (88·6)	0	140 (100)	0
Feb 2009	29	14 (48·2)	0	14 (100)	0
Mar 2010	81	60 (74·1)	0	60 (100)	0
Feb 2011	16	15 (93·8)	0	15 (100)	0
Mar 2012	90	79 (87·8)	0	76 (96·2)	3
Total	574	488 (85·0)	41	444 (91·0)	3

a Numbers in brackets represent the proportion of group B RSV‐positive samples that were sequenced of all those that were identified and subtyped as group B.

b Numbers in brackets represent percentage of the sequenced group B genotypes that genotyped into BA.

Peak epidemic month given by the three letter month abbreviation.

The three patients were admitted on March 1, on March 23, and on May 16, 2012, and were aged 14·5, 1·7, and 5·5 months, respectively, at the time of admission. Two of the patients were admitted with severe pneumonia, and the third patient had very severe pneumonia (see ref.^11^ for syndromic definitions). All three nucleotide sequences (KEN/201/Mar/2012, KEN/202/Mar/2012 and KEN/203/May/2012) obtained from the three patients were identical across the entire 633 nucleotides sequenced.

A blast search to track their ancestry (http://blast.ncbi.nlm.nih.gov/Blast.cgi) querying the second hypervariable region of G gene found that these three non‐BA sequences were closely related to strains from Brazil collected in between 2004 and 2007 with the top matches being those isolated in 2007. These Brazilian 2007 strains were non‐BA type and have been classified under the GB3 genotype in their GenBank annotation.

**Figure 1:**
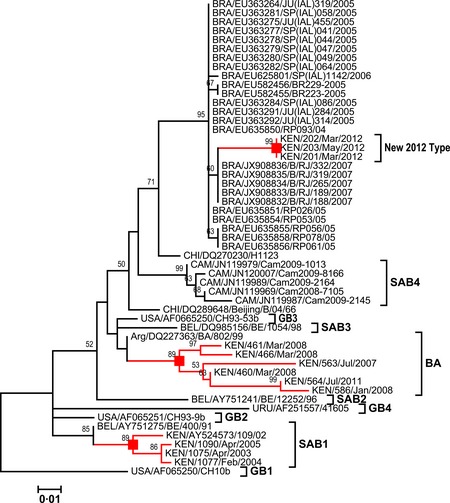
A maximum‐likelihood tree of 53 sequences including the top 25 blast hits, the 3 new non‐BA Kilifi sequences, 10 sequences representative strains previously collected at Kilifi (both BA and non‐BA), and 15 representative sequences of all the previously described group B genotypes. The tree is derived from an alignment of the carboxy terminus region nucleotide sequences of the RSV G protein gene [264‐nt‐long region (324 for BA)]. The nodes of branches with exclusively Kilifi sequences are indicated with a red‐filled square box, and the associated branches of these Kilifi sequences are also colored in red. The nomenclature of the taxa is a three‐letter code for the country of sequence origin/GenBank accession number/strain name.

The phylogenetic relationship of the Kilifi 2012 non‐BA sequences, the other group B genotypes identified at Kilifi (BA and SAB1), the top 25 of the blast hits, and representative sequences of all previously described group B genotypes around the world is given in Figure 1. The Kilifi 2012 non‐BA sequences and the previously circulating non‐BA sequences at Kilifi (SAB1) fell on separate branches that were distant from each other. The Kilifi 2012 non‐BA sequences and the non‐BA sequences that were isolated in Cambodia (2008–2009, designated SAB4^9^) and China (before 2005) alongside the Brazilian sequences (2004–2007) fell into one cluster but with only moderate support (bootstrap value 50%) although clearly separated from the other group B RSV genotypes, Figure 1.

The average between‐group genetic distances between the Kilifi 2012 non‐BA sequences and the Brazilian, Chinese, and Cambodian non‐BA viruses were 2·8%, 6·4%, and 9·4%, respectively. These distances imply significant diversification between these sequences despite the relatively close relationship on the tree. The MRCA analysis predicted the Kilifi 2012 non‐BA viruses and that the Brazilian viruses diverged from June 2007 (95% HPD, January 2002–February 2012), while the divergence with the Cambodian 2008‐2009 non‐BA viruses was predicted to have occurred much earlier – August 2000 (95% HPD, February 1985–May 2009). However, these estimates should be interpreted with caution as the HPD confidence intervals are very wide and overlapping.

**Figure 2:**
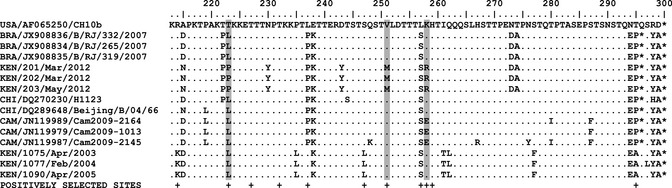
The figure shows an alignment of partial G‐protein‐predicted amino acid sequences for the Brazilian, Chinese, and Cambodian non‐BA sequences that were identified in the second half of the last decade and the Kilifi non‐BA sequences [both the new 2012 and the old (SAB1 strains)]. The sequences are compared with GB1 strain (USA/CH10b) given at the top of the alignment as a reference strain. Where the amino acid sequence is similar to the GB1 strain sequence, the position is shown with dot along the column otherwise implies a mutation. The asterisks denote stop codons. At the bottom of the columns, positions previously identified as positively selected in group B RSV are indicated with a plus (+) symbol.^13^, ^14^ Gray‐highlighted columns show amino acid sequence positions that changed and were also identified to be positively selected. The taxa nomenclature is a three‐letter code for the country of sequence origin/GenBank accession number/strain name.

**Figure 3:**
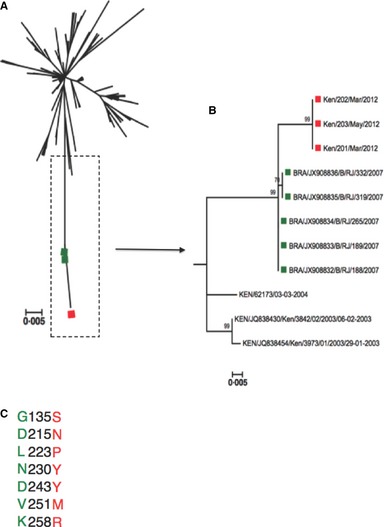
Panel (A) shows a maximum‐likelihood tree from 232 sequences comparing 599 nucleotide positions of the RSV G protein gene. This includes all unique BA sequences observed at Kilifi between 2003 and 2012 (224 sequences), the 3 Kilifi 2012 non‐BA strains (marked red), and 5 of the close Brazilian non‐BA sequences collected in 2007 that span this length (marked green). In the alignment used to generate this tree, the 60‐nucleotide insertion of the BA strains was removed to investigate whether the remaining nucleotide positions were highly similar to BA, which could then suggest that the new non‐BA strains emerged by spontaneous deletion of this region from BA genotype. However, we observe that these new Kilifi non‐BA strains cluster far away on the tree from the current BA strains and are close to the Brazilian non‐BA strains – boxed in (a) with a dashed rectangle and expanded as panel (B). Panel (C) shows the seven amino acid changes in the Kilifi 2012 non‐BA strains from the Brazilian 2007 strains and their respective positions that they were observed (six of the seven changes occur in the second hypervariable region).

Within the carboxy terminus region of the encoded G protein, the Brazilian 2007 viruses and the Kilifi 2012 non‐BA viruses differ by six amino acids, Figures 2 and 3. Interestingly, three of these changes affect sites that were previously shown to be under positive selection in group B RSV,^13^, ^14^ that is, positions 223, 251, and 258. Studies with RSV A strains have previously noted that multiple epitopes lie in this region of the RSV G gene recognized by human convalescent sera, and changes affecting some particular positions, some of which were identified as positively selected, can have profound effect on the overall antibody recognition.^3^ Thus, these substitutions in the 2012 non‐BAs could be of considerable significance.

We have previously reported the detection of minority genomes within two patients sampled in 2008 that showed a 60‐nucleotide deletion at the exact position where the insertion occurred in the BA variant, and we speculated this as a potential mechanism by which strains without the duplication could re‐emerge.^15^ Our phylogenetic analysis comparing the new non‐BA strains with the circulating BA strains at Kilifi from which the 60‐nucleotide duplication sequence was excised found the new strains were only distantly related to BA; that is, they do not represent BA strains that have recently lost their duplication (Figure 3). This observation of wild‐type non‐BA viruses still in circulation provides a mechanism for replacement of the BA strains if the BA selective advantage diminishes.

In conclusion, we report the total dominance of the BA genotype at Coastal Kenya among group B isolates over six epidemics and re‐appearance of non‐BA strains during the first half of 2012. These new non‐BA strains appear to have been circulating globally, albeit as a minority genotype, for several years and are not emerging *de novo* from the BA genotype. Compared with their closest relatives in GenBank, the new strains were found to have undergone changes at positions believed to be located in immune epitopes, and it will be interesting to observe whether these variants will increase in prevalence over time and succeed in dominance over the BA genotype. These observations raise questions on the amount of undetected RSV diversity existing and the mechanisms driving changes to population‐level RSV diversity.

## GenBank accession numbers

The G sequences of the Kilifi strains reported in this study are deposited into GenBank under the accession numbers KC263040–KC263051.
